# The Relationship between Body Weight Change and Body Constitutions of Traditional Chinese Medicine in Patients with Schizophrenia

**DOI:** 10.1155/2016/9585968

**Published:** 2016-10-13

**Authors:** Jui-Fen Cheng, Xuan-Yi Huang, Te-Le Liu, Ruey-Yun Wang, Han-Yi Ching

**Affiliations:** ^1^School of Nursing, China Medical University, Taichung, Taiwan; ^2^Department of Nursing, China Medical University Hospital, Taichung, Taiwan; ^3^Department of Nursing, National Taipei University of Nursing and Health Sciences, Taipei, Taiwan; ^4^Nursing Department, TsaoTun Psychiatric Center, Ministry of Health and Welfare, Nantou, Taiwan; ^5^Department of Public Health, China Medical University, Taichung, Taiwan; ^6^Psychiatry Department, TsaoTun Psychiatric Center, Ministry of Health and Welfare, Nantou, Taiwan

## Abstract

*Objective*. To explore the relationship between body constitution (BC) types and weight change in patients with schizophrenia and who underwent second-generation antipsychotics (SGAs) treatment.* Method*. Body weight and waist circumference of eighty-five participants were measured for 6 consecutive weeks. Constitutions of Yin-Xu, Yang-Xu, and Stasis were assessed using the Body Constitution Questionnaire (BCQ).* Results*. Participants with body constitutions Yin-Xu (50.6%), Yang-Xu (49.4%), or Stasis (38.8%) exhibited worse physical condition and unhealthy daily habits, particularly in Stasis constitution. Moreover, Stasis constitution was significantly associated with several factors, including BMI, body weight, waist circumference, perception of stress, perception of health, staying up late, and less physical exercise. However, perception of stress showed significant difference in Yin-Xu, Yang-Xu, and Stasis. Generalized estimating equation (GEE) analysis revealed that significant time effects in body weight increase in the imbalanced BC types and gentleness BC type. SGAs induced weight gain in imbalanced BC type as well as gentleness BC type, especially treated with olanzapine.* Conclusions*. This is the first study to explore the longitudinal relationship between BC and weight gain in schizophrenia patients undergoing SGAs treatment. Health care providers should focus on weight gain problems in schizophrenia patients who underwent SGAs treatment.

## 1. Introduction

Schizophrenia is a chronic, disabling illness that frequently relapses, particularly when treatment is discontinued. Therefore, improving medication adherence has been emphasized as a key component of the management of schizophrenia [1]. Second-generation antipsychotics (SGAs) are now widely used in the treatment of schizophrenia because of their clinical efficacy and low incidence of neurological side effects. Previous studies have reported several advantages of SGAs over first-generation antipsychotics (FGAs), including lower relapse rates, lower overall treatment failure, lower hospitalization rates, and greater improvement of the side effects of acute extrapyramidal and tardive dyskinesia [2–4]. However, some patients, particularly those who were younger and those with a lower baseline body mass index, who underwent SGAs treatment experience significant weight gain [5]. Being overweight or obese may adversely affect a patient's physical health and self-esteem, often resulting in discontinuation of medication [6].

There is growing concern regarding metabolic side effects such as weight gain and insulin resistance and syndromes such as dyslipidemia and type 2 diabetes mellitus [7–10]. Currently, there is virtually no way of predicting who will be affected by antipsychotic-induced weight gain and identifying individuals at a higher risk of the same. The human constitution has been extensively researched in oriental as well as Western medicine for a long time. For example, Hippocrates identified four types of substances (blood, phlegm, choler, and black bile); Lee classified humans into four Sasang constitutional types in Korea [11, 12]. The most commonly used methods for classifying constitutions in traditional Chinese medicine (TCM) include the Nine-Constitution Scale developed by the China Association for Traditional Chinese Medicine [13, 14] and the Body Constitution Questionnaire (BCQ) developed by Su. Previous studies have used BCQ to measure body constitution (BC) as they are reliable and valid [15–17]. In TCM philosophy, human beings exhibit vulnerability to particular diseases based on their individual BC, making classification of personal constitutions important. Although previous studies used BCQ to examine the effects of TCM body constitutions in pregnant women and type 2 diabetics, there are no studies that focused on mental illnesses [18, 19].

TCM has been practiced for more than 2000 years, and the application and acceptability of TCM as a prominent alternative to modern medicine have increased in Western countries [20]. This longitudinal study is the first to explore the association between BC types and weight changes in patients with schizophrenia in Taiwan. Examination of individual constitution will allow the identification of appropriate diet, exercise, herbal therapy, acupressure, and massage therapies for the achievement of Yin-Yang balance, health promotion, and disease prevention [21, 22]. Therefore, the aim of this study was to explore the relationship between BC types and body weight change in patients with schizophrenia and who underwent SGA treatment in the acute ward.

## 2. Materials and Methods

### 2.1. Participants

Several criteria were established to ensure that the participants were appropriate for the study. These included participants who had a diagnosis of schizophrenia, according to International Classification of Diseases, Ninth Revision, Clinical Modification (ICD-9-CM, code 295) criteria and participants over 20 years of age and receiving SGAs treatment at the acute ward in the Area Psychiatry Medical Net Core Hospital in central Taiwan between June 2014 and May 2015 (*N* = 90). Patients who exhibited substance abuse and are not able to understand or answer the questions were excluded from this study. Of these, a total of 85 participants completed the study (completion rate: 94%). Ethical approval was granted by the ethics committee of the Institutional Review Board of the Tsaotun Psychiatric Center, Ministry of Health and Welfare, which is the leading psychiatric hospital in central Taiwan. The participants were informed of the details of the study and given the option of withdrawing at any point. Signed informed consent forms were collected from all study participants prior to commencement of the study.

### 2.2. Data Collection

This study combines a cross-sectional and longitudinal study design to further clarify the effect of TCM body constitution on weight gain in patients treated with SGAs. Demographic data (age, sex, age at the time of diagnosis, perception of stress (0–10), perception of health (0–100), and habits including smoking, drinking, sleeping, and physical activity) and body constitution were collected using a questionnaire. After participating in this study for six weeks, all of the participants completed a structured questionnaire by face-to-face interview. The participants' body weight (while wearing light clothing and no shoes) and waist circumference (at the end of a normal expiration) were measured in the morning immediately after the patient woke up and emptied his/her bladder for 6 consecutive weeks. Body weight in kilograms was measured using a calibrated platform scale (K-120; Nagata). Body mass index was calculated by dividing each participant's body weight (kg) by the square of their height (m^2^). The participants' blood pressures were measured in the seated positions and rest for at least 15 minutes in the morning every day. The participants' laboratory data were collected from medical records, which were detected on the next day of hospitalization, according to the hospitalized admission routine of the acute ward. Medications data were collected from medical records. Physicians did not participate in this study; however, they prescribed the SGAs for the participants based on their clinical experience.

The BCQ used in this study was developed by Su and consists of 44 items on a 5-point Likert-type response scale, ranging from 1 (never happened) to 5 (always happens) [15, 23]. The total score ranged between 44 and 220, and patients were classified into balanced BC type or one or more of three imbalanced BC types, as follows: Yin-Xu (19 items, scores ranging from 19 to 95), Yang-Xu (19 items, scores ranging from 19 to 95), and Stasis (16 items, scores ranging from 16 to 80). Participants who did not reach the threshold scores for the three imbalanced BC types were considered as balanced or gentleness type. Coexistence of multiple imbalanced BC types was possible. According to previous studies, BCQ is a reliable and valid tool for evaluating body constitution. Cronbach's *α* coefficients were 0.85, 0.88, and 0.88 for Yang-Xu [15], Yin-Xu [16], and Stasis [17], respectively. The intraclass correlation coefficients were greater than 0.7 for most items [15–17].

## 3. Results

**Figure 1:**
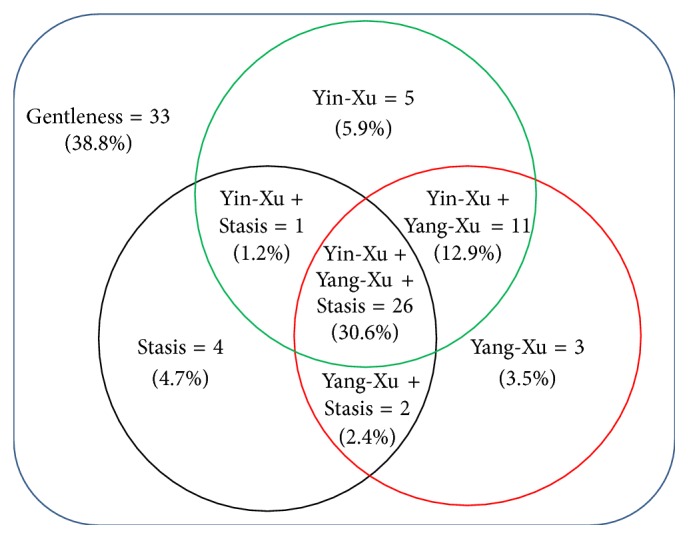
Distribution of body constitution types.

This study recruited 90 schizophrenic patients, including 41 males and 44 females completing this study. Four participants withdrew as they were discharged and one expressed reluctance to continue participation in the study. The numbers of participants' comorbidity diseases such as diabetes mellitus, hypertension, hyperlipidemia, HBsAg (positive), and anti-HCV (positive) were 6 (7.1%), 2 (2.4%), 2 (2.4%), 16 (18.8%), and 7 (8.2%), respectively.  Table 1 compares the basic characteristics and biomarkers between patients with and without Yin-Xu, Yang-Xu, and Stasis. Of the 85 participants, the average age and duration of schizophrenia were 43.0 years (SD = 11.3 years) and 19.2 years (SD = 11.4 years), respectively. The number of patients classified as Yin-Xu, Yang-Xu, and Stasis was 43 (50.6%), 42 (49.4%), and 33 (38.8%), respectively. The number of the participants with each body constitution type and the corresponding percentage distribution are shown in Figure 1. Gentleness constitution was observed in up to 38.8% of the participants. A majority of the patients (*N* = 40, 47.1%) exhibited two to three unbalanced body constitution types simultaneously.

Patients with body constitution types Yin-Xu, Yang-Xu, or Stasis were older and had longer duration of schizophrenia, negative perceptions of health, and worse physical condition than those without Yin-Xu, Yang-Xu, or Stasis. Patients with Yin-Xu exhibited significantly higher mean values of perception of stress and amylase than those without Yin-Xu. Patients with Yang-Xu showed significantly higher mean values of duration of schizophrenia, triglycerides, and perception of stress and significantly lower mean values of perception of health than those without Yang-Xu. Patients with Stasis exhibited significantly higher mean values of body weight, waist circumference, body mass index, and perception of stress and significantly lower mean values of self-perception of health than those without Stasis (Table 1).

Table 2 lists the crude odds ratios for association factors in Yin-Xu, Yang-Xu, and Stasis. The Stasis constitution was significantly associated with worse physical condition including BMI (OR, 1.11; 95% CI, 1.02–1.22), body weight (OR, 1.03; 95% CI, 1.00–1.06), waist circumference (OR, 1.04; 95% CI, 1.01–1.07), perception of stress (OR, 1.16; 95% CI, 1.01–1.33), and perception of health (OR, 0.98; 95% CI, 0.96–1.00) and poor daily habits including staying up late (OR, 2.56; 95% CI, 1.02–6.39) and less physical exercise (OR, 6.61; 95% CI, 1.28–34.14; OR, 4.49; 95% CI, 1.17–17.23). Multiple backward logistic regression analysis after adjustment for history of illness, body weight, body mass index, waist circumference, self-perception of health, self-perception of stress, triglycerides, staying up late, and exercise showed that the factors correlated with Stasis constitution were duration of schizophrenia (OR, 1.06; 95% CI, 1.01–1.12), body mass index (OR, 1.16; 95% CI, 1.05–1.29), and self-perception of stress (OR, 1.20; 95% CI, 1.02–1.41).

**Figure 2:**
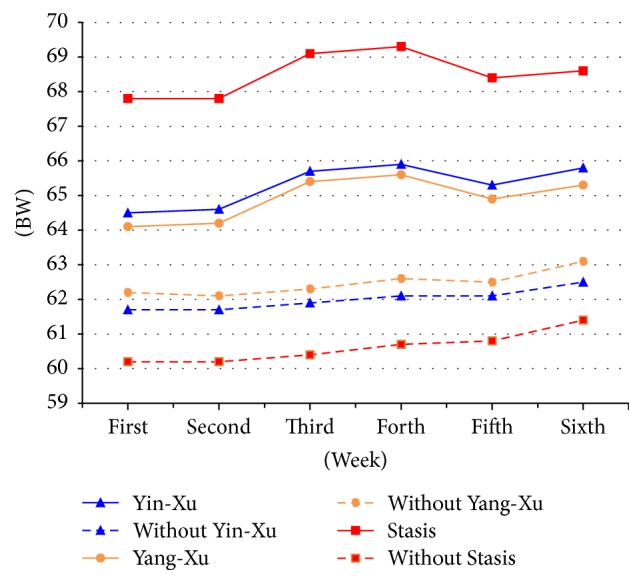
Change of body weight between different body constitutions within six weeks.

A general estimating equation (GEE) was used to analyze differences in change in body weight between BC groups over the period of 6 consecutive weeks [24]. There were no significant differences in body weight increase with the Yin-Xu (from 64.53 to 65.82) and without Yin-Xu (from 61.72 to 62.51) groups over the 6-week duration. However, significant time effects were observed (Figure 2 and Table 3). For the Yin-Xu group, the mean body weight increased significantly over the 6-week period, with the mean weight in the fifth (65.34 ± 2.39, *p* < 0.05) and sixth (65.82 ± 2.39, *p* < 0.005) weeks being significantly higher than that in the first week (64.53 ± 2.44) (Figure 2). For the group without Yin-Xu, a significant difference in mean body weight increase was only observed between the sixth (62.51 ± 2.32, *p* < 0.05) and first (61.72 ± 2.31) week (Figure 2).

There were no significant differences in body weight increase with the Yang-Xu (from 64.14 to 65.34) and without Yang-Xu (from 62.17 to 63.06) groups over the 6-week period. However, significant differences in time effects were observed between the two groups (Figure 2). The mean body weight increased significantly over the 6 weeks in the Yang-Xu group, with the fifth (64.93 ± 2.54, *p* < 0.05) and sixth (65.34 ± 2.54, *p* < 0.005) weeks being significantly higher than the first (64.14 ± 2.58, *p* < 0.05) week (Figure 2). For the group without Yang -Xu, significant differences in mean body weight increase were observed only between the sixth (63.06 ± 2.18, *p* < 0.05) and the first (62.17 ± 2.18) week (Figure 2). There were no significant differences in body weight increase in the combined Yin-Xu, Yang-Xu, and Stasis group (from 65.62 to 66.64) and without combination groups (from 62.06 to 63.11) over the 6-week period, although significant differences in time effects were seen (Table 3). The mean body weight increased significantly over 6 weeks in the group without Stasis, with the fifth (60.75 ± 1.78, *p* < 0.05) and sixth (61.36 ± 1.79, *p* < 0.005) weeks being significantly higher than the first week (60.17 ± 1.79). In the Stasis group, no significant differences in mean body weight increase were observed over the 6 weeks. However, the Stasis group exhibited significantly higher body weight than the group without Stasis in the first, second, third, fourth, and fifth weeks. There were no significant differences in body weight increase between the combined Yin-Xu, Yang-Xu and Stasis group (from 65.62 to 66.64) and without combination groups (from 62.06 to 63.11) over the 6-week period, although significant differences in time effects were seen (Table 3). The mean body weight increased significantly over 6 weeks in the group without combination, with the fifth (62.58 ± 1.83, *p* < 0.05) and sixth (63.11 ± 1.82, *p* < 0.01) weeks being significantly higher than the first week (62.06 ± 1.81). In the combined Yin-Xu, Yang-Xu, and Stasis group, no significant differences in mean body weight increase were observed over the 6 weeks.

The number of participants treated with different types of SGAs is as follows: risperidone (*N* = 33), clozapine (*N* = 17), olanzapine (*N* = 16), zotepine (*N* = 10), amisulpride (*N* = 5), quetiapine (*N* = 3), and paliperidone (*N* = 1). Besides the SGAs, the other medications are estazolam (*N* = 16 (18.8%)) and clonazepam (*N* = 1 (1.2%)).

The paired* t*-test was used to compare the first week to the second, third, fourth, fifth, and sixth weeks in risperidone (*N* = 33), clozapine (*N* = 17), olanzapine (*N* = 16), zotepine (*N* = 10), amisulpride (*N* = 5), and quetiapine (*N* = 3).  Table 4 showed a significant increase difference with the fourth, fifth, and sixth weeks in olanzapine (*p* = 0.05, 0.012, and 0.003, resp.) and a significant decrease difference with the second weeks in risperidone (*p* = 0.042). Furthermore, there were no significant differences between different body constitutions treated with different types of SGAs.

## 4. Discussion

TCM focuses on preventive medicine, which is basically aimed at improving health.

Yin and Yang are two of the most fundamental concepts of TCM as they are the foundation of diagnosis and treatment. The fundamental pathology of onset, progression, and variations in disease lies in the disturbance of the balance between Yin and Yang [25], and preserving this balance can lead to good health. “Yang” consists of the energy required for maintaining body function, and Yang-Xu (Yang deficiency) implies a decrease in energy levels during physiological functioning of the body [23]. Yang deficiency is a chronic syndrome characterized by cold limbs; inability to feel warm; profuse clear urine; lack of sexual desire; pale face, tongue, and lips; a slow, weak pulse; and so forth. “Yin” consists of all material aspects of the body such as blood and body fluids, and Yin-Xu (Yin deficiency) implies decreasing a person's materials required for maintenance or achievement of body function [16, 26]. The common symptoms of Yin-Xu include depleted moistening action of the body, leading to symptoms such as hot flushes, dry throat, heat in the “five palms” (palms, soles, and chest), night sweats, and hard stool. “Stasis” is said to result in Qi stagnation, phlegm, and blood stasis and is formed by persistent disruption and less efficient dynamic interaction between Yin and Yang caused by any external or environmental stimuli (e.g., stressful events or climate change) [17]. Individuals with this body constitution may express physical symptoms and signs such as tingling pain in the limbs, dizziness, and chest tightness.

To the best of our knowledge, this is the first study to focus on the body constitution of patients with schizophrenia. There is a higher prevalence of imbalanced body constitutions (Yin-Xu, Yang-Xu, and Stasis) in people with schizophrenia (50.6%, 49.4%, and 38.8%, resp.) compared with people with type 2 diabetes (27.8%, 12.9%, and 12.9%, resp.) [19]. Our findings are consistent with previous study, which divides schizophrenia into six types as follows: (1) internal disturbance of pyrophlegm, (2) internal retention of phlegm-damp, (3) Qi stagnation and blood stasis, (4) Yin deficiency and hyperactivity of fire due to Yin deficiency, (5) Yang deficiency, and (6) others [27, 28]. In our study, patients with Yin-Xu, Yang-Xu, or Stasis constitutions reported poor physical condition and self-perception of health and significantly higher levels of stress compared with patients without these constitutions in the same population. Previous studies examining different populations have also reported that people classified as having imbalanced BC types exhibited significantly poorer Health-Related Quality of Life (HRQOL) than those classified as having gentleness BC type [19, 29]. In addition, patients with Yin-Xu, Yang-Xu, or Stasis in our study were overweight (BMI > 24). There is a higher prevalence of obesity in people with Stasis than in those with gentleness BC type, and these patients are also at a higher risk of metabolic syndromes, central obesity (waist circumference, 90.5 ± 15.2), and significantly lesser physical exercise (Tables 1 and 2). Stasis frequently occurs in long-term chronic illnesses and is an important pathology of many disease processes in TCM. Previous studies have demonstrated that Stasis constitution generally exerts the greatest negative effects on HRQOL [19, 29]. Some studies have also reported that Phlegm-Stasis constitutions are induced by hyperlipidemia and atherosclerosis [30, 31]. The triglyceride levels in patients with Yang-Xu constitutions were significantly higher than in those without Yang-Xu. Patients with Stasis constitutions in our study reported fasting glucose and triglyceride levels that were higher than in those without Stasis, but this was not statistically significant. The lack of statistically significant differences in biomarkers between the Stasis and without Stasis groups may have been a result of the sample size and limited number of biomarkers measured in this study. Future studies should explore body constitution-related biomarkers such as high-density lipoproteins, low-density lipoproteins, and Ankle Brachial Index (ABI) to aid the modernization of TCM. In our study, patients with schizophrenia suffered from mental restlessness as well as psychological unrest induced by disharmonious Yin-Yang. To achieve a perfectly balanced life, it is important to maintain the balance between body, mind, and soul.

There were no statistically significant time–group interactions in body weight increase. However, a significant time–effect in body weight increase was observed in groups with or without of Yin-Xu, Yang-Xu, and Stasis. Furthermore, the Stasis group exhibited significantly higher body weight than the group without Stasis in the first, second, third, fourth, and sixth weeks. In our study, SGAs induced weight gain in imbalanced BC types as well as gentleness BC type. This has also been demonstrated by several meta-analyses, of which one included 307 articles and demonstrated that all antipsychotics resulted in weight gain over time [32–34]. Despite the lack of statistically significant time-group interactions, patients with disharmonious body constitutions (Yin-Xu, Yang-Xu, or Stasis) exhibited higher body weight than those without Yin-Xu, Yang-Xu, or Stasis. Furthermore, the change in body weight in patients with disharmonious constitutions and treated with SGAs exhibited fluctuations, with increases in the third and fourth weeks and decreases in the fifth week. Patients without Yin-Xu, Yang-Xu, or Stasis showed smooth and slight increases in body weight upon treatment with SGAs. Our study findings differed from those of a previous study, which reported that the greatest amount of weight gain occurred within the first weeks of treatment [5]. Furthermore, this was not limited to patients who had already gained a significant amount of weight, particularly those with Stasis constitutions [35]. Previous studies suggest that monitoring and controlling weight gain within the first 6 weeks of treatment are crucial as losing weight after 6 weeks is difficult [5]. Awareness in this area is essential for monitoring and preventing increases in body weight. Monitoring, evaluation, and nonpharmacological interventions have been previously suggested to prevent weight gain and metabolic risk [36–38]. This study highlights the fact that schizophrenic patients with disharmonious body constitutions exhibit unhealthy lifestyle habits, stress, and obesity. Previous studies demonstrate that an unhealthy lifestyle with reduced physical activity, sleep disturbances, and poor diet selection impacts physical and mental health, which is similar to our findings [39, 40]. Further studies are required to confirm the results of our studies.

Despite its contributions, this study has several limitations. Firstly, the longitudinal design of this study included a follow-up period of only 6 weeks. Future studies should involve a longer follow-up period such as 6 months to allow detection of the relationship between body constitution and body weight. Secondly, the sample size used in this study was only 85, and future research should increase this number and explore related biomarkers such as high-density lipoproteins, low-density lipoproteins, and ABI. Lastly, the sample in this study was selected from a psychiatric hospital in central Taiwan, limiting generalization of the results to other populations.

We recommend that future studies should explore the longitudinal relationship between health interventions, transformation of body constitutions, and change in body weight (e.g., exercise).

## 5. Conclusion

To the best of our knowledge, this study is the first to explore the longitudinal relationship between body constitution and weight gain in patients with schizophrenia undergoing SGA treatment. In our study, patients with imbalanced body constitutions reported poorer physical condition and unhealthy daily habits than those with gentle constitutions, specifically those with Stasis constitutions. SGAs induced weight gain in imbalanced BC type as well as gentleness BC type.

However, there was no significant increase in body weight in imbalanced BC type compared with gentleness BC type in this study. However, there is strong evidence of the association between SGAs and accelerated weight gain. Health care providers should pay more attention to the problem of weight gain in people with schizophrenia, specifically those with Stasis constitutions. These findings may serve as a valuable reference for healthcare professionals providing health promotion for mental illness care and also as empirical evidence for use in future intervention-based research.

## Figures and Tables

**Table 1:** Comparisons of schizophrenia characteristics and biomarkers between constitutions of Yin-Xu, Yang-Xu, and Stasis (*N* = 85).

Variable	Total (*N* = 85)	Gentleness (*N* = 33)	Yin-Xu			Yang-Xu			Stasis		
			No (*N* = 42)	Yes (*N* = 43)	*p* value	No (*N* = 43)	Yes (*N* = 42)	*p* value	No (*N* = 52)	Yes (*N* = 33)	*p* value
Age (yr)	43.0 ± 11.3	41.2 ± 11.6	41.9 ± 12.0	44.1 ± 10.6	0.38	40.9 ± 11.7	45.1 ± 10.6	0.09	42.7 ± 11.6	43.4 ± 10.9	0.77
Duration of schizophrenia (yr)	19.2 ± 11.4	16.8 ± 9.7	17.1 ± 10.7	21.3 ± 11.8	0.88	16.6 ± 10.3	21.9 ± 11.9	0.03^*∗*^	17.6 ± 11.0	21.7 ± 11.8	0.11
BMI (Kg/m^2^)	23.9 ± 5.2	23.0 ± 4.7	23.8 ± 5.3	24.8 ± 4.9	0.39	23.8 ± 5.3	24.9 ± 4.9	0.12	23.3 ± 4.5	26.0 ± 5.7	0.03^*∗*^
Body weight (Kg)	63.1 ± 15.7	60.9 ± 13.0	62.5 ± 15.2	65.8 ± 15.9	0.33	63.1 ± 14.5	65.3 ± 16.7	0.50	61.3 ± 13.0	68.6 ± 18.2	0.04^*∗*^
Sleeping time (hr)	9.0 ± 1.4	9.0 ± 1.2	9.0 ± 1.2	9.0 ± 1.5	0.82	9.1 ± 1.5	8.9 ± 1.2	0.58	8.8 ± 1.1	9.3 ± 1.6	0.17
Waist circumference (cm)	84.5 ± 15.0	81.4 ± 13.4	83.14 ± 14.1	87.1 ± 15.0	0.21	82.7 ± 14.1	87.7 ± 14.9	0.12	81.7 ± 13.3	90.5 ± 15.2	0.006^*∗∗*^
Perception of stress (0–10)	3.8 ± 3.3	1.8 ± 2.2	2.5 ± 2.7	5.1 ± 3.3	0.00^*∗∗*^	2.7 ± 3.1	5.0 ± 3.1	0.00^*∗∗*^	3.2 ± 3.3	4.8 ± 3.1	0.03^*∗*^
Perception of health (0–100)	71.7 ± 23.2	80.0 ± 17.9	74.5 ± 23.5	68.9 ± 22.8	0.27	78.1 ± 19.1	65.1 ± 25.3	0.01^*∗∗*^	76.4 ± 21.8	64.3 ± 23.7	0.02^*∗*^
Fasting glucose (mg/dL)	95.4 ± 23.6	92.6 ± 20.4	94.5 ± 24.3	96.2 ± 23.1	0.75	94.1 ± 24.4	94.2 ± 18.2	0.99	92.3 ± 18.0	97.2 ± 26.1	0.31
Triglycerides (mg/dL)	116.2 ± 70.1	98.0 ± 47.3	110.4 ± 67.9	121.8 ± 74.0	0.46	97.2 ± 44.1	135.6 ± 86.8	0.01^*∗∗*^	112.9 ± 72.7	121.3 ± 68.6	0.6
Cholesterol (mg/dL)	170.7 ± 36.1	166.0 ± 29.2	167.7 ± 31.1	176.0 ± 38.0	0.88	167.3 ± 31.5	176.5 ± 37.6	0.98	176.0 ± 35.6	165.5 ± 32.9	0.31
Hemoglobin	13.6 ± 2.6	16.2 ± 17.4	13.3 ± 1.3	14.0 ± 3.5	0.22	13.2 ± 1.4	14.1 ± 3.4	0.11	13.6 ± 3.2	13.7 ± 1.4	0.96
Amylase	56.0 ± 28.1	49.1 ± 19.7	49.9 ± 18.7	61.9 ± 34.1	0.04^*∗*^	52.6 ± 20.5	59.4 ± 34.1	0.83	55.2 ± 24.6	57.1 ± 33.3	0.76
SBP (mmHg)	121.3 ± 17	122.4 ± 18.1	122.0 ± 18.6	120.6 ± 15.9	0.71	120.8 ± 17.6	121.7 ± 17.0	0.81	121.3 ± 16.7	121.2 ± 18.2	0.98
DBP (mmHg)	83.0 ± 12.7	82.9 ± 12.2	83.3 ± 11.8	82.8 ± 13.8	0.86	82.7 ± 12.2	83.3 ± 13.4	0.83	82.2 ± 12.6	84.4 ± 13.0	0.44

Date were presented as mean ± SD; ^*∗*^ *p* < 0.05; ^*∗∗*^ *p* < 0.01.

BMI: body mass index; SBP: average of systolic blood pressure; DBP: average of diastolic blood pressure.

Duration of schizophrenia (yr) was calculated from the time of first onset and at the time of diagnosis of schizophrenia.

Sleeping time: from going to bed until waking up, excluding interruption time.

**Table 2:** Logistic regression model: odds ratios for association factors in constitution of Yin-Xu, Yang-Xu, and Stasis (*N* = 85).

Variable	Yin-Xu			Yang-Xu			Stasis		
	OR	95% CI	*p* value	OR	95% CI	*p* value	OR	95% CI	*p* value
Duration of schizophrenia (yr)	1.03	(0.99–1.08)	0.104	1.05	(1.00–1.09)	0.030^*∗*^	1.03	(0.99–1.98)	0.100
BMI (Kg/m^2^)	1.03	(0.95–1.11)	0.503	1.04	(0.95–1.13)	0.417	1.11	(1.02–1.22)	0.019^*∗*^
Body weight (Kg)	1.01	(0.98–1.04)	0.404	1.01	(0.98–1.04)	0.557	1.03	(1.00–1.06)	0.033^*∗*^
Waist circumference (cm)	1.02	(0.99–1.05)	0.264	1.02	(0.99–1.05)	0.181	1.04	(1.01–1.07)	0.013^*∗*^
Perception of stress (0–10)	1.31	(1.12–1.53)	0.001^*∗∗*^	1.26	(1.09–1.46)	0.002^*∗∗*^	1.16	(1.01–1.33)	0.003^*∗∗*^
Perception of health (0–100)	0.99	(0.97–1.01)	0.266	0.97	(0.95–0.99)	0.013^*∗*^	0.98	(0.96–1.00)	0.024^*∗*^
Triglycerides (mg/dL)	1.00	(1.00–1.01)	0.459	1.01	(1.00–1.01)	0.019^*∗*^	1.00	(1.00–1.01)	0.591
Staying up late	1.80	(0.73–4.44)	0.202	2.40	(0.96–6.00)	0.061	2.56	(1.02–6.39)	0.045^*∗*^
Exercise									
Sometimes	0.94	(0.22–3.92)	0.93	1.16	(0.27–4.93)	0.84	6.61	(1.28–34.14)	0.024^*∗*^
Usually	2.05	(0.72–5.85)	0.182	2.53	(0.87–7.39)	0.89	4.49	(1.17–17.23)	0.028^*∗*^

^*∗*^ *p* < 0.05,  ^*∗∗*^ *p* < 0.01.

Staying up late: sleeping after midnight.

Exercise (sometimes: 1-2 times/week; usually: more than 2 times/week).

**Table 3:** General estimating equation for comparison of body weights between constitutions of “Yin-Xu,” “Yang-Xu,” “Stasis,” with “Yin-Xu, Yang-Xu and Stasis” within the study's time frame (*N* = 85).

Variables	Yin-Xu				Yang-Xu				Stasis				Combined Yin-Xu, Yang-Xu, and Stasis			
	Beta	SE	Wald *χ* ^2^	*p* value	Beta	SE	Wald *χ* ^2^	*p* value	Beta	SE	Wald *χ* ^2^	*p* value	Beta	SE	Wald *χ* ^2^	*p* value
Intercept	61.72	2.31	713.41	0.00^*∗∗*^	62.16	2.18	823.90	0.00^*∗∗*^	60.17	1.76	1165.93	0.00^*∗∗*^	62.06	1.81	1169.78	0.000^*∗∗*^
Group (no as reference)	2.82	3.36	0.70	0.40	1.98	3.38	0.34	0.56	7.65	3.63	4.42	0.035^*∗*^	3.57	4.0664	0.769	0.381
Time (week 1 as reference)																
Week 2	−0.05	0.16	0.09	0.76	−0.081	0.1561	6.73	0.60	0.008	0.1610	0.002	0.962	−0.01	0.14	0.005	0.943
Week 3	0.16	0.33	0.24	0.62	0.130	0.3291	10.06	0.69	0.075	0.207	0.832	0.717	0.024	0.19	0.016	0.899
Week 4	0.36	0.36	0.98	0.32	0.344	0.3622	5.68	0.34	0.294	0.3228	1.381	0.240	0.27	0.22	1.44	0.231
Week 5	0.35	0.30	1.35	0.25	0.377	0.3055	3.97	0.22	0.579	0.2931	3.899	0.048^*∗*^	0.52	0.26	3.96	0.047^*∗*^
Week 6	0.80	0.33	5.81	0.016^*∗*^	0.895	0.3453	6.73	0.01^*∗∗*^	1.190	0.3227	13.603	0.000^*∗∗*^	1.05	0.29	13.67	0.000^*∗∗*^
Group × time (week 2)	0.096	0.24	0.16	0.69	0.167	0.24	0.489	0.485	0.38	0.58	0.41	0.52	0.02	0.26	0.005	0.946
Group × time (week 3)	0.073	0.42	0.03	0.86	0.134	0.42	0.101	0.751	0.36	0.48	0.55	0.46	0.16	0.40	0.16	0.69
Group × time (week 4)	0.192	0.49	0.15	0.70	0.232	0.49	0.225	0.635	1.41	1.32	1.14	0.29	0.22	0.52	0.17	0.678
Group × time (week 5)	0.455	0.49	0.88	0.35	0.411	0.49	0.710	0.399	1.36	1.10	1.54	0.22	0.17	0.59	0.08	0.781
Group × time (week 6)	0.493	0.54	0.82	0.36	0.302	0.55	0.304	0.581	1.36	1.10	1.54	0.22	−0.03	0.66	0.003	0.959

^*∗*^ *p* < 0.05;  ^*∗∗*^ *p* < 0.01.

**Table 4:** Paired *t*-test of body weight change in different types of SGAs.

	Risperidone (*N* = 33)	Clozapine (*N* = 17)	Olanzapine (*N* = 16)	Zotepine (*N* = 10)	Amisulpride (*N* = 5)	Quetiapine (*N* = 3)
Pair 1 2nd-1st	−0.32 ± 0.88^*∗*^	0.12 ± 1.38	0.36 ± 1.03	0.30 ± 1.48	0.32 ± 1.02	−0.40 ± 0.53
Pair 2 3rd-1st	−0.38 ± 1.52	0.06 ± 1.52	0.68 ± 1.67	0.58 ± 1.65	0.56 ± 1.46	−0.67 ± 1.15
Pair 3 4th-1st	−0.37 ± 1.59	0.41 ± 1.83	1.09 ± 2.05^*∗*^	1.11 ± 2.27	1.04 ± 2.94	−0.60 ± 1.04
Pair 4 5th-1st	−0.27 ± 1.93	0.62 ± 2.18	1.44 ± 2.00^*∗*^	1.49 ± 2.64	1.16 ± 3.59	−0.17 ± 0.29
Pair 5 6th-1st	0.16 ± 2.23	0.94 ± 2.57	2.03 ± 2.31^*∗*^	1.75 ± 2.50	2.22 ± 3.64	—

Date were presented as mean ± SD; ^*∗*^ *p* < 0.05.
